# A Routine ‘Top-Down’ Approach to Analysis of the Human Serum Proteome

**DOI:** 10.3390/proteomes5020013

**Published:** 2017-06-06

**Authors:** Arlene M. D’Silva, Jon A. Hyett, Jens R. Coorssen

**Affiliations:** 1 Department of Molecular Physiology, The Molecular Medicine Research Group, School of Medicine, Western Sydney University, Campbelltown, NSW 2150, Australia; A.Dsilva@westernsydney.edu.au; 2 Department of High Risk Obstetrics, RPA Women and Babies, Royal Prince Alfred Hospital, Sydney, NSW 2050, Australia; Jon.Hyett@sswahs.nsw.gov.au; 3 Faculty of Graduate Studies, and the Departments of Health Sciences and Biological Sciences, Brock University, St. Catharines, ON L2S 3A1, Canada

**Keywords:** deep Imaging, Lithium Dodecyl Sulfate, prefractionation, postfractionation, proteomics, proteoforms, three-dimensional gel electrophoresis, two-dimensional gel electrophoresis

## Abstract

Serum provides a rich source of potential biomarker proteoforms. One of the major obstacles in analysing serum proteomes is detecting lower abundance proteins owing to the presence of hyper-abundant species (e.g., serum albumin and immunoglobulins). Although depletion methods have been used to address this, these can lead to the concomitant removal of non-targeted protein species, and thus raise issues of specificity, reproducibility, and the capacity for meaningful quantitative analyses. Altering the native stoichiometry of the proteome components may thus yield a more complex series of issues than dealing directly with the inherent complexity of the sample. Hence, here we targeted method refinements so as to ensure optimum resolution of serum proteomes via a top down two-dimensional gel electrophoresis (2DE) approach that enables the routine assessment of proteoforms and is fully compatible with subsequent mass spectrometric analyses. Testing included various fractionation and non-fractionation approaches. The data show that resolving 500 µg protein on 17 cm 3–10 non-linear immobilised pH gradient strips in the first dimension followed by second dimension resolution on 7–20% gradient gels with a combination of lithium dodecyl sulfate (LDS) and sodium dodecyl sulfate (SDS) detergents markedly improves the resolution and detection of proteoforms in serum. In addition, well established third dimension electrophoretic separations in combination with deep imaging further contributed to the best available resolution, detection, and thus quantitative top-down analysis of serum proteomes.

## 1. Introduction

Detailed analyses of the serum proteome are important as they provide a source of diagnostic or prognostic biomarkers as well as insight into the mechanisms underlying disease development and progression [1,2]. Due to the heterogeneity of disease, single protein markers are frequently not sufficiently predictive of a condition to be of significant clinical value. A panel of candidate biomarkers is typically needed to improve diagnostic efficacy [3,4]. Although fitness-for-purpose must be considered in deciding between the use of bottom-up or top-down proteomic approaches [5], quantification of disease-associated alterations is often best achieved by the latter, in which intact proteoforms (i.e., protein species) are resolved from complex biological samples using techniques such as 2-dimensional gel electrophoresis (2DE) coupled with mass spectrometry (MS) [6]. 2DE is the only available proteomic technique that can simultaneously resolve hundreds-to-thousands of proteoforms in a single analytical run, while also enabling multiple parallel analyses. As the only such routine top-down analytical protocol, it is thus the only available approach that enables quantitative profiling of large sets of complex protein mixtures; that is, as part of the routine analytical protocol, this approach resolves protein isoforms, splice variants, and the vast range of post-translationally modified protein species that define biological functionality.

Serum contains one of the most complex proteomes that has thus far been researched. The dynamic range of protein characteristics (e.g., isoelectric point, mass, hydrophobicity, concentration, and post-translational modifications) makes effective coverage of the serum proteome very challenging as it is difficult to resolve such a diverse range of macromolecules [7]. High-abundance proteins tend to mask those of lower abundance and have typically been removed to allow better resolution of other species [8]. However, removal of this fraction risks removal of non-targeted proteins that may impact on our understanding of the mechanisms underlying disease as well as on discovery and quantification of novel biomarkers [9]. In addition, removal of the most abundant proteins merely exposes a second cohort that is highly abundant in comparison to other species—so this intervention fails to resolve the fundamental problem of dynamic range and largely obviates the objective of quantitative analysis [5].

As with other complex samples, methods used to reduce the complexity of the serum proteome are based on the physicochemical and structural characteristics of the constituent proteins, including solubility, hydrophobicity, molecular weight and isoelectric point. Ultracentrifugation provides a simple approach for the separation of high molecular weight proteins but is non-selective and thus also does not address the issue of protein-protein binding and non-specific losses [10,11]. Similarly trichloroacetic acid (TCA) has been used to precipitate high abundance proteins such as albumin by forming a TCA-albumin complex [12]. Phase separation of detergents such as Triton X-114 (TX-114) distinguishes between proteins on the basis of hydrophobicity and is relatively cheap and versatile but the partitioning behaviour depends on the properties of the proteins being resolved (e.g., molecular weight and surface exposure of different amino acid residues) and may still not fully address the issue of non-specific protein losses to one fraction or the other [13]. Other methods are mainly used to target the removal of hyper-abundant proteins and are based on affinity phases, ion-exchange and antigenic activity [14]; these processes are similarly compromised by a lack of specificity and/or by the potential of complex protein-protein interactions leading to the unintended removal of (lower abundance) species bound to the highly abundant fraction. Whilst these techniques facilitate identification of some less abundant proteins there is a risk that others will not be recognised and that any attempts at quantification do not in fact represent the native state of the proteome.

**Figure 1:**
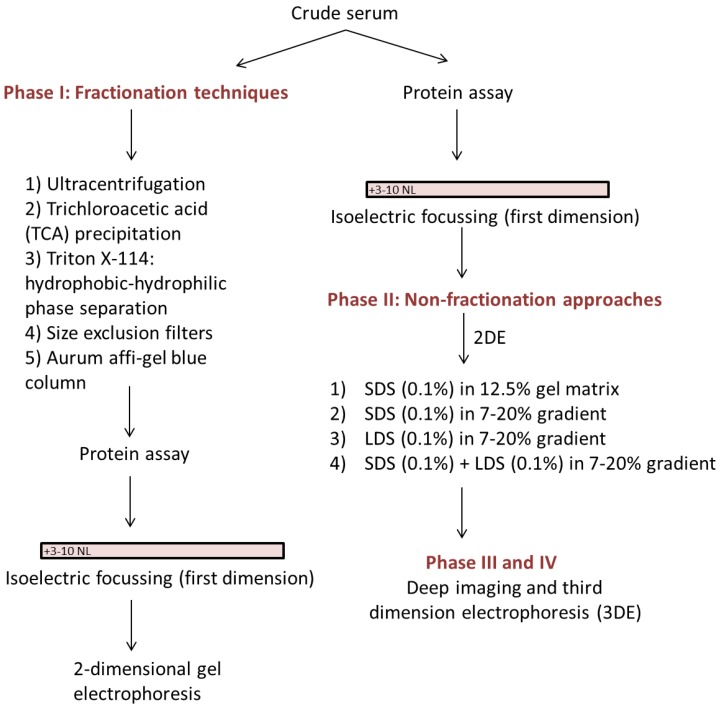
A simplified flow chart of various phases used during optimization of serum two-dimensional gel electrophoresis (2DE).

In preparation for assessment of the serum proteomes of pregnant women who laboured preterm, we have developed an analytic technique that does not remove protein species but nonetheless enables improved differentiation of both high and low abundance proteoforms, of both high and low molecular weight. As the whole serum proteome is conserved, the technique also allows quantification of species, and for further future improvements as detection methods continue to improve in sensitivity and selectivity [15,16,17,18,19]. In addition to the various techniques discussed and tested in order to optimize efficient resolution of the native serum proteome, we have also combined a robust and well-established 2DE protocol [20,21] with (i) a new, high sensitivity staining and detection protocol [15,17,18]; (ii) postfrationation or third dimension electrophoresis (3DE) [22,23]; and (iii) Deep Imaging [23,24,25]—as well as selective staining to assess phospo- and glycoprotein subproteomes (i.e., proteoforms) in order to extract as much information as possible from each gel [9,24] (Figure 1). 3DE is used to further resolve co-migrating proteins that appear as hyper-abundant spots after initial resolution by 2DE, using a gradient gel customized to provide optimal resolution within the target molecular weight range [22,23]. Deep imaging involves excising saturating spots/regions and imaging the gel at 750 V, thus enabling detection of lower abundance proteins [23,24,25]. We thus report the development of an efficient, sensitive and reproducible technique that substantially improves the quantitative protein profiling of native human serum, and that should prove widely applicable to a range of comparable sample types including plasma and urine.

## 2. Materials and Methods

Banked serum samples were analysed from a cohort of women attending for combined first trimester screening, a screening test for Down syndrome at 11–13^+6^ weeks’ gestation. Samples were collected between 28 June 2011 and 15 April 2013, and were initially centrifuged for 10 min at 2000× *g* in a NATA approved clinical laboratory within four hours of collection. These samples were used to determine serum PaPP-A and free βhCG levels, while the residual serum was immediately stored at −80 °C. The subsequent pregnancy outcomes have been recorded and the samples selected for this work were from uncomplicated pregnancies. Sample use was approved by the Royal Prince Alfred Hospital ethics committee (X11-0305/HREC/11/RPAH/472). A reference proteome was created using serum pooled from three samples.

### 2.1. Protein Assay

Protein estimation was performed using the EZQ Protein Quantitation Kit with BSA standards according to the manufacturer’s instructions (Molecular Probes, Eugene, OR, USA). A baseline native serum profile was prepared by solubilising crude serum in 2DE buffer containing 8 M urea, 2 M thiourea, 4% (*w*/*v*) CHAPS and a cocktail of protease, kinase and phosphatase inhibitors (referred to as PI) at a ratio of 1× conc of PI (initial conc of 500×): 2 mL of serum [26].

#### 2.1.1. 2-Dimensional Gel Electrophoresis (2DE)

Proteins were resolved using a well-established protocol and further detail is provided in the supplementary methods section [24]. Several methods of sample preparation prior to the first dimension (termed phase I) and at the 2DE stage (termed phase II) were tested for potential improvements in resolution whilst ensuring minimal loss of low abundant species and preservation of proteoform integrity for quantitative assessment.

**Phase I**: Testing of fractionation techniques involved use of Ultracentrifugation, Trichloroacetic acid (TCA) precipitation, Triton X-114 phase separation, Size exclusion filters, and the Aurum Affi-Gel Blue column prior to the first dimension of resolution (Figure 1).

#### 2.1.2. Ultracentrifugation

Considering the previously identified complement of membranous material and membrane protein in serum and plasma [27,28], ultracentrifugation was carried out according to Churchward et al., (2005) (supplementary methods) [29]. 2 mL of thawed serum with an added 1× conc of PI was centrifuged at 146,542× *g* for three hours at 4 °C using a Beckman Coulter Optima L-100 XP ultracentrifuge; the separate supernatant and pellet fractions were collected.

#### 2.1.3. Trichloroacetic Acid (TCA) Precipitation

500 µL TCA (100% (*w*/*v*)) was added to 500 µL of crude serum and incubated at −30 °C overnight (supplementary methods) [12]. The sample was centrifuged at 15,000× *g* for 30 min at 4 °C.

#### 2.1.4. Triton X-114: Hydrophobic-Hydrophilic Phase Separation

TX-114 phase separation was carried out using a modification of the Bordier protocol [30]. In brief, a cushion of 2000 µL of 6% (*w*/*v*) sucrose, 10 mM Tris-HCl (pH 7.4), 150 mM NaCl and 0.5% TX-114 was placed at the bottom of a Falcon 15 mL conical centrifuge tube. 500 µL of crude serum sample with 1 × PI was then overlaid on this cushion and the tube was incubated for 3 min at 30 °C to effect condensation. The tube was centrifuged at RT for 3 min at 300× *g* to effect phase separation, yielding a clear, viscous lower detergent phase (DP) and an upper aqueous phase (AP). After phase separation, the DP and AP were analysed separately as described in the supplementary material.

#### 2.1.5. Size Exclusion Filters

100 kDa and 50 kDa Amicon ultra-centrifugal low protein binding filter units (Merck Millipore, Billerica, MA, USA) were briefly rinsed with 200 µL of 0.9% NaCl before use. 2 mL of crude serum was mixed with an equal volume of saline containing 1× PI, and centrifuged in two stages (using the 100 kDa and 50 kDa filters, respectively) to produce three fractions of nominally > 100 kDa (fraction A), 50–100 kDa (fraction B) and < 50 kDa (fraction C); both centrifugation steps were carried out at 1008× *g* for 20 min at 4 °C. Desalting and the estimation of protein concentration (both as above) were carried out and all three fractions were then analysed by 2DE.

#### 2.1.6. Aurum Affi-Gel Blue Column

The effect of albumin removal on analysis of the serum proteome was also tested using Aurum affi-gel blue mini columns (Bio-Rad, Hercules, CA, USA). This protocol has been explained in the supplementary material.

**Phase II:** Optimisation of ‘non-fractionation’ approaches involved using serum in the native form for the first dimension while replacing or supplementing SDS with LDS for the second dimension (Figure 1).

#### 2.1.7. Lithium Dodecyl Sulfate (LDS) vs. Sodium Dodecyl Sulfate (SDS)

We explored an alternative strategy to improving the resolution of protein species by resolving native serum on large (i.e., 20 cm × 20 cm) 7–20% gradient acrylamide gels which enabled a larger amount of protein to be resolved. LDS alone as well as a combination of LDS and SDS were tested by first resolving native serum without any fractionation on 2D gels. LDS was added in the equilibration buffer (6 M Urea, 20% glycerol, 375 mM Tris; pH 8.8, 0.1% LDS or a combination of 0.1% LDS and 0.1% SDS) when reducing and alkylating the IPG strip after IEF, as well as in the gel matrix (7% and 20% acrylamide, 375 mM Tris, 0.1% LDS or a combination of 0.1% LDS and 0.1% SDS) used in the second resolving dimension (see supplementary material).

**Phase III:** Deep imaging and third-dimension electrophoresis (3DE).

Here, 2DE was combined with 3DE (a postfractionation approach) and integrated with a well-established ‘deep imaging’ protocol [23,24,25]. For each 2DE analysis, 500 µg of protein was resolved on 17 cm, 3–10 NL IPG strips, as described above; areas of near-saturating signal (i.e., hyper-abundant proteins) were manually excised from the cCBB stained gels using a scalpel. Imaging was repeated at 750 V for higher sensitivity protein detection. All excised spots were further resolved using a standardized 3DE protocol. Briefly, those gels designated for 3DE were initially stained using the reversible zinc-imidazole protocol [24], and saturating spots/regions were excised, the zinc fixation reversed on both the main gel and the excised regions, and the latter were turned 90° relative to the initial path of resolution and then subjected to a third round of electrophoresis, on narrow gradient gels customized to the corresponding molecular weight region; 10–15% for heavy cut spots corresponding to 60–200 kDa and 15–18% for light cut spots corresponding to 10–50 kDa. Once electrophoresis was completed, gels were stained with cCBB, destained, and imaged as described above.

### 2.2. Phospho and Glyco Staining

Pro-Q Diamond and Pro-Q Emerald stains were used to identify post translation modifications (PTM), specifically, phosphorylation and glycosylation, respectively. Pro-Q Emerald 488 Glycoprotein and Pro-Q Diamond Phosphoprotein stains were obtained from Life Technologies (Carlsbad, CA, USA). Following 2DE, phospho- and glyco- staining was carried out on control serum sample according to manufacturer’s protocols.

### 2.3. Image and Statistical Analyses

The resulting resolved protein spots from all 2DE and 3DE gels were quantitatively analysed using automated spot detection in Delta 2D (version 4.08; DECODON GmbH, Greifswald, Germany). For fractionation techniques, all images of replicate gels of both fractions were fused to create a representative image. When serum was resolved, without any fractionation, mean spot counts have been reported. Only protein species consistently and reproducibly detected across a given set of replicate gels were considered for the spot count. These spot counts were obtained excluding the gel edges and the protein ladder [24]. In order to examine the variation in protein spot numbers following fractionation and alternate methods, the analysis of variance (ANOVA) with Tukey’s Multiple Comparison Test was carried out.

## 3. Results

The mean spot count for the preliminary reference proteomes of native serum was 367 ± 2 for mini gels and 709 ± 15 for large gels (Table 1 and Table 2); this is ‘standard’ SDS-PAGE (2% SDS in equilibration buffer and 0.1% SDS in resolving gel matrix) in the second dimension [29]. As well established in the literature, larger gels with larger protein loads resulted in better resolution of proteoforms (*p* ≤ 0.001).

For the purpose of presentation, total protein species detected using each approach are given as mean ± SEM (standard error of the mean) of the technical replicates. Individual mean ± SEM for every fraction type are reported as supplementary data.

**Figure 2:**
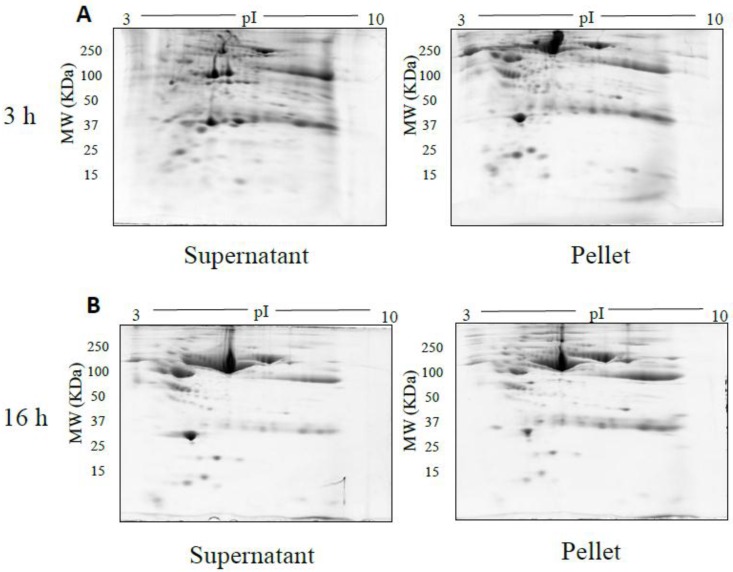
Representative gel images of supernatant and pellet fractions obtained after 3 h (**A**), 16 h (**B**) ultracentrifugation respectively. 100 µg of supernatant and pellet fractions was loaded onto a 7 cm 3–10 NL IPG strip, following 2DE on 12.5% acrylamide gel.

**Figure 3:**
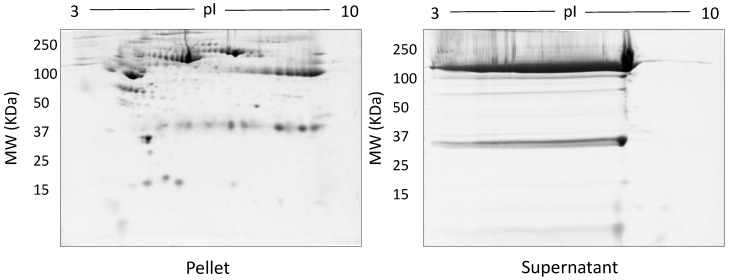
Representative gel images following TCA/acetone precipitation: Shown are the pellet and supernatant fractions resolved on 12.5% acrylamide gels using 100 µg of fractionated serum.

**Figure 4:**
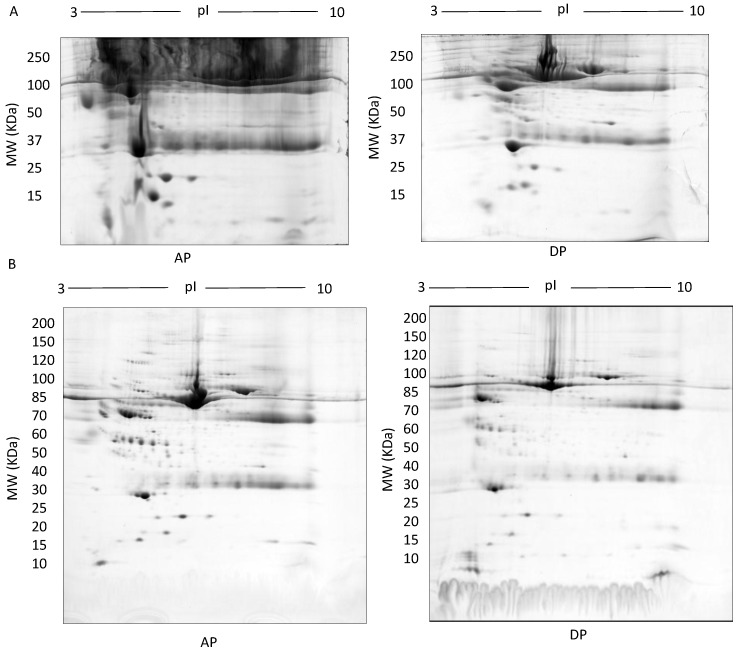
**TX-114:** Representative images of gels of the detergent phase (DP) and aqueous phase (AP) (**A**), 500 µg fractionated serum resolved on large 7–20% gradient gels after multiple wash steps to ensure removal of TX-114 detergent (**B**).

**Figure 5:**
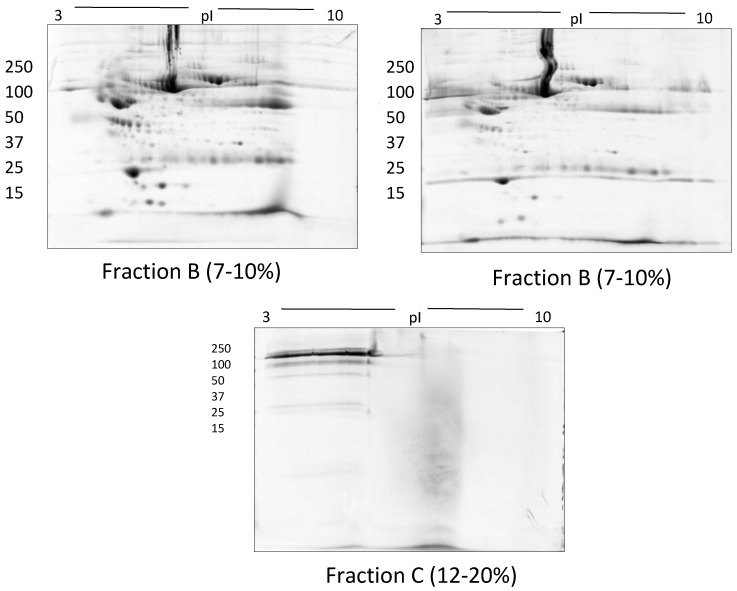
Representative gel images of > 100 kda (fraction A) and flow through fractions i.e., 50–100 kda (fraction B) and < 50 kda (fraction C) following the use of Amicon size exclusion filters.

**Figure 6:**
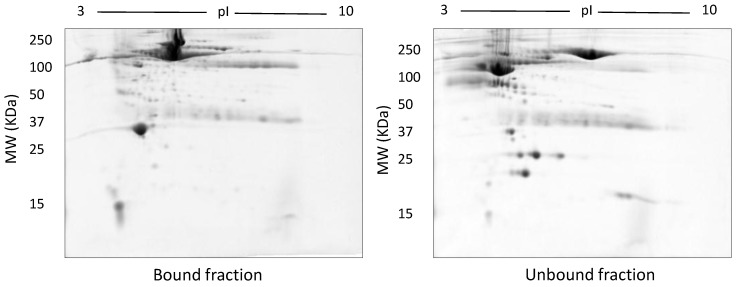
Representative gel images of bound and unbound fractions collected following use of the Bio-Rad Affi-Gel kit. Following manufacturer’s instructions, 100 µg of both bound and unbound fractions were resolved on 12.5% acrylamide gels after undergoing isoelectric focusing on a 3–10 NL IPG strip.

The spot counts and representative 2DE gels for the different methods of fractionation carried out in phase I optimisation are shown in Table 1 and Figure 2, Figure 3, Figure 4, Figure 5 and Figure 6, respectively. All fractionation methodologies showed poor protein separation in terms of the molecular properties being targeted. That is, there was considerable overlap in the distribution of protein species between fractions that would have been expected to be far more distinct or defined based on the fractionation technique used. The overlap between the fractions has been represented in the form of fusion images in the supplementary material section. Ultracentrifugation (either 3 h or 16 h) was used to pellet a denser protein fraction. The resolved 2DE gels indicated that a substantial portion of albumin was found in the pellet fraction but most protein species were distributed across both the soluble and pellet fractions (Figure 2). Following TCA/acetone precipitation of serum, the resulting 2DE gels of the pellet fraction were better resolved to a certain extent; there was less streaking and a lower background than seen after ultracentrifugation (Figure 3) and some less abundant species previously masked by albumin were better resolved. However, the total number of protein species detected was reduced compared to the native serum proteome (Table 1), and gels of the organic supernatant fraction suggested that substantial amounts of proteins and charge variants other than albumin were lost using this protocol.

In TX-114 phase separation, the AP did not provide effective separation of proteins, apparently due to residual TX-114 (Figure 4). Incorporating multiple wash steps of the AP helped to remove remaining TX-114 (Figure 4). The number of proteins resolved in AP and DP using TX-114 phase separation was found to be significantly increased (*p* ≤ 0.001) compared to native serum (Table 1). Though size exclusion filters performed well in promoting the overall resolution of proteins species relative to the native serum gel, they did not appear to enable effective separation of protein species of different size ranges, nor in limiting albumin to a single fraction. There was thus also substantial evidence that low molecular weight proteins were retained in the high MW (>100 kda) fraction (Figure 5). The commercial affinity columns also proved to be poor discriminators, removing a large number of non-specifically bound proteins together with albumin (Figure 6). Overall, for these initial 12.5% mini gel tests, 3 h ultracentrifugation and TX-114 precipitation resulted in a significant increase in protein species detected compared to native serum. Moreover, when resolved on a large gradient gel of 7–20%, prior TX-114 phase separation resulted in the subsequent detection of 779 ± 51 spots in comparison to 709 ± 15 (*p* ≤ 0.001) detected when analysing unfractionated native serum (Table 1).

**Figure 7:**
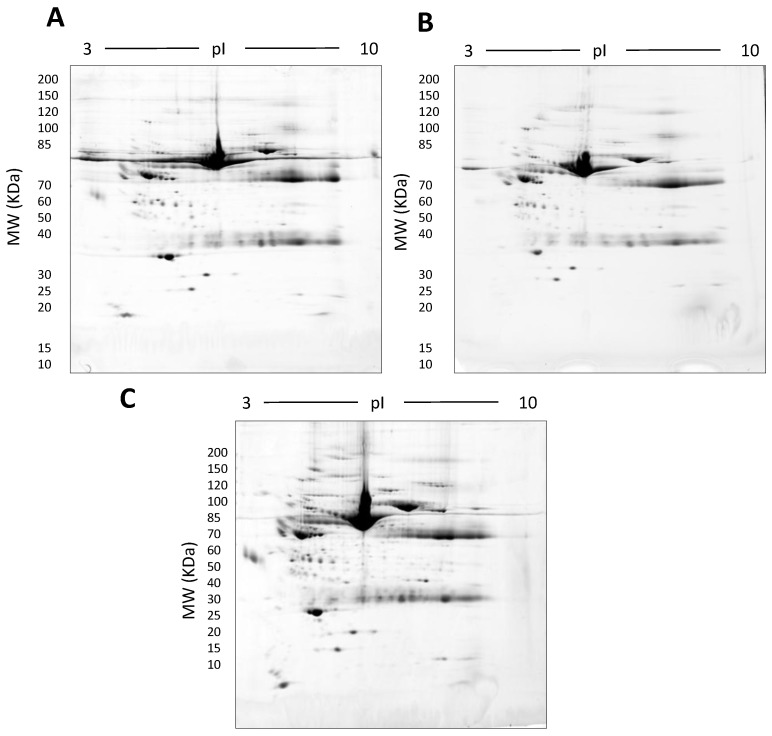
Representative gel images showing the effect of various detergents on the resolution of unfractionated serum: 500 µg total protein, 17 cm, 3–10 NL IPG, and 7–20% second dimension gradient gel using 0.1% SDS (**A**), 0.1% LDS (**B**) and 0.1% SDS + 0.1% LDS (**C**).

**Figure 8:**
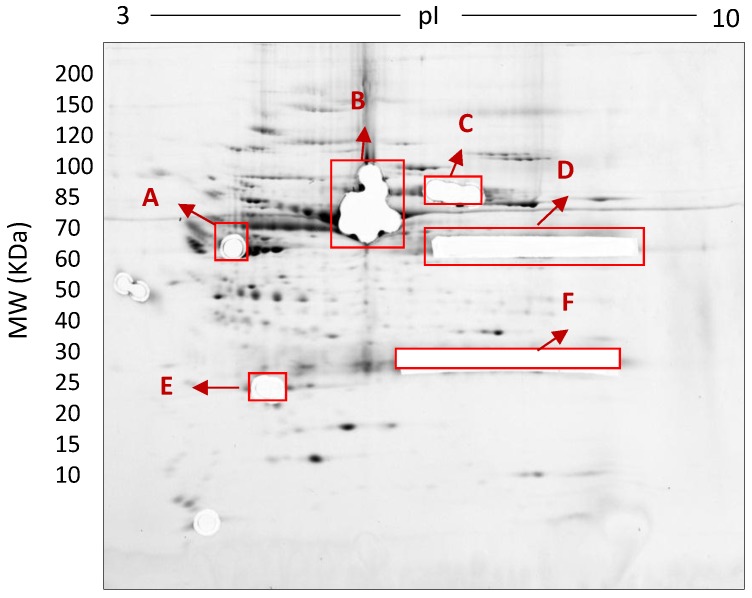
Deep imaged serum proteome after excision of high abundance proteins (i.e., saturating spots). 500 µg total protein, 17 cm, 3–10 NL IPG, and 7–20% second dimension gradient gel using 0.1% SDS + 0.1% LDS.

**Figure 9:**
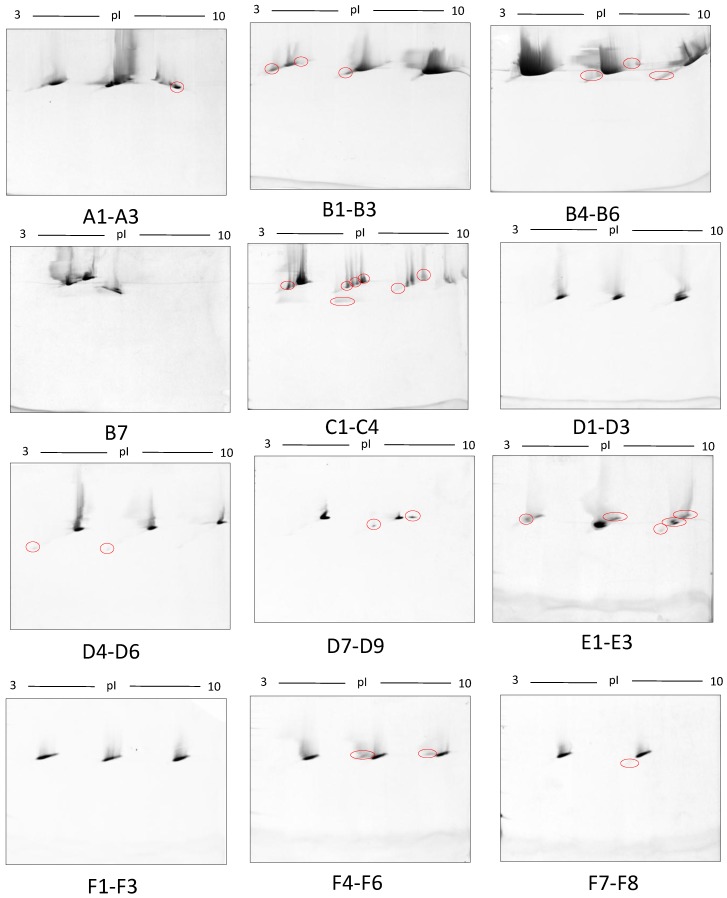
Third-dimension separations of high-density (i.e., hyper-abundant) protein regions excised from serum proteomes resolved by 2DE; red circles indicate protein species resolved from co-migrating hyper-abundant proteins. Designations A-F refer to excised gel regions (see Figure 8), and in each case the associated numbers refer to specific subsections of those excised gel pieces (i.e., A1-A3 means excised region A was subdivided into three approximately equal sized gel pieces that were then resolved in parallel on third gels (see Materials and Methods).

In this early stage of optimisation (i.e., phase I), most of the 2DE was carried out on mini gels. However, there was a significant increase in detectable protein species resolved on 7–20% gradient gels rather than 12.5% gels in the second dimension (Table 1 and Table 2, and Figure 7). Hence phase II optimisation was carried out on large, 7–20% gradient gels. Phase II optimisation involved resolving native serum without any fractionation step. Table 2 documents a significant increase in the number of protein spots resolved by using a combination of SDS (0.1%) and LDS (0.1%) as compared to the classical SDS (0.1%) in the second dimension (Figure 7). Furthermore, deep imaging of the 0.1% SDS + 0.1% LDS gels resulted in a greater than 2-fold increase of resolved protein species compared to the standard SDS alone (*p* ≤ 0.001) (Table 2, Figure 8). Third dimension separation of hyper-abundant protein spots (particularly those known to correspond to albumin, immunoglobin heavy and light chains, and serotransferrin) from serum proteomes initially resolved by 2DE enabled resolution of additional protein species from these more abundant co-migrating proteins (Figure 9). When fully resolved by 3DE separation, the largest 2DE ‘spot’ (i.e., more of an irregular shaped, saturating ‘blotch’) was found to consist of more than one protein, clearly signifying that multiple species are present in large and/or poorly resolved ‘spots’ (i.e., those not relatively small and tightly circular; Figure 9) [22,24].

**Figure 10:**
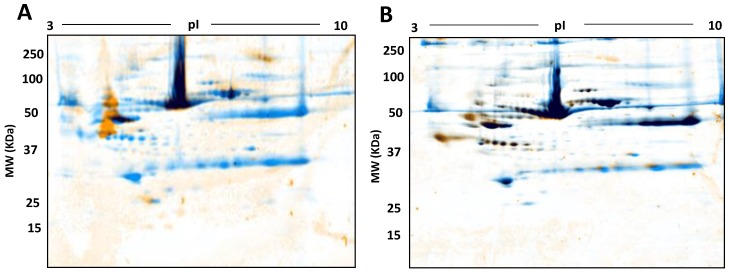
Phospho- and Glyco- proteome assessment: Control serum proteomes resolved by 2DE, stained for phosphoproteins (**A**) and glycoproteins (**B**) respectively followed by total staining by cCBB. In A and B, orange represents phosphoproteins and glycoproteins identified by Pro-Q Diamond Phosphoprotein and Pro-Q Emerald 488 Glycoprotein stains, blue represents total proteins identified by cCBB and black represents the overlap of phosphoproteins and glyco proteins with the total proteins.

In addition to the general optimisation of serum analyses, we were also interested in PTM. For confirmation at this stage, we thus also trialled the use of phospho- and glyco- staining as a first analysis of select proteoforms (Figure 10).

## 4. Discussion

A variety of prefractionation methods have been developed to remove albumin and other high abundance proteins from serum samples prior to proteomic analysis thereby presumably enhancing the detection of lower abundance species of potential interest. However, this approach could lead to the concomitant removal of some non-targeted proteins of potential scientific and clinical interest. We have developed a routine technique for analysing the proteome of crude serum samples that does not involve fractionation and therefore retains the native complement and stochiometry of protein species, and fully enables quantitative analysis. Through systematic comparison of a variety of techniques, we have shown that relatively heavy loading with 500 µg total serum protein on 17 cm 3–10 NL IPG strips in the first dimension followed by second dimension resolution on 7–20% gradient gels with a combination of LDS and SDS detergents provides the optimal current top-down methodology to resolve the human serum proteome. Our optimisation process was carried out in three phases (Figure 1). Phase I optimisation (fractionation) was carried out to reduce the complexity of serum by restricting highly abundant proteins to one fraction. All of the commonly used prefractionation techniques (phase I) that we tested showed substantial overlap in terms of protein content in the separate fractions. This overlap indicates insufficient resolving power of these methods and increases concerns that quantitative analysis using any of these methods is unlikely to yield satisfactory outcomes. In phase II, we were able to obtain significantly increased numbers of resolved protein species by using crude serum itself (Table 2 and Figure 7); this involved optimising 2DE without any fractionation and hence no loss of proteins, in order to facilitate subsequent quantitative assessment. In addition to using standard 2DE techniques for analysis we found that phase III, 3DE of highly abundant proteins, enabled further resolution of co-migrating proteoforms, and Deep Imaging enabled further detection of lower abundance species (Table 2, Figure 8 and Figure 9) [17,23,24]. Use of selective phospho- and glycoprotein stains confirmed the well-established ability of 2DE to routinely resolve proteoforms (Figure 10).

Whilst serum markers play an important role in medical screening, many have seemingly been discovered somewhat serendipitously rather than through a systematic process of biomarker discovery. The subsequent translation of biomarkers from discovery to clinical practice involves multiple stages with many potential pitfalls. One major challenge is the inherent biological complexity of the serum proteome [7]. To date, the majority of research directed to identifying serum biomarkers through a top-down 2DE approach has involved the use of depletion columns to remove the most abundant proteins. The traditional depletion strategy involves the use of a hydrophobic dye, Cibacron blue, which has a high affinity for albumin. This strategy for removing albumin is frequently used in proteomic analyses of serum because of its relatively low cost [31]. The use of immunoaffinity media, which consist of matrices with covalently attached antibodies to the most abundant proteins has also become commonplace [32,33]. Antibody-based affinity chromatography techniques to remove albumin have been established to isolate and investigate albumin-bound proteins [34]. Immunoglobulins G represent the second most abundant protein species in serum and some methods have been established to remove not only albumin, but also this class of proteins [35,36]. Methodologies based on the depletion of high abundance proteins followed by liquid chromatography-mass spectrometry have been used as well [37,38]. Combinatorial hexapeptide ligand libraries have also been used to enable detection of low abundance proteins of interest [39,40]. This approach also works on the principle of affinity chromatography and has been coupled with antibody based depletion methods to treat human serum and other complex biological extracts [32,33,41,42]. However, a major a disadvantage of this method is that in spite of the large number of ligands, if none has affinity for a given protein, the latter will not be captured [39]. Furthermore, quantitative removal of any given species is not ensured, and nonselective loss of proteins has also been documented, indicating similar selectivity issues and concerns for later quantitative analyses as noted for other affinity-based fractionation approaches [8,43,44]. Overall, the methods above are based on depletion of proteins to ‘reduce’ the complexity of the serum proteome, usually with the aim of qualitatively detecting more proteins rather than quantitatively identifying and confirming any given species as a biomarker.

However, these depletion strategies can lead to the concomitant non-specific removal of proteins that may be of potential interest [8]. As a transport protein, albumin binds to various compounds including hormones, lipids and amino acids so the loss of low-abundance peptides or small proteins of interest, such as cytokines, is inevitable [45]. Stempfer et al., (2008) quantified the effectiveness of human high-abundance serum and plasma protein depletion using 2DE and bottom-up shotgun MS (i.e., 2D capillary chromatography with MS/MS). The data indicate that some low-abundance proteins were still identified following the depletion protocol; nevertheless, on resolving the depleted fractions, several proteins were found to adhere to the depletion matrix and were thus completely lost to analysis [46]. In brief, these methods have a clear shortcoming in terms of the loss of potentially critical protein species that could be of translational significance in a clinical setting and/or to understanding disease mechanisms, and thus also hamper or even obviate the quantitative analyses necessary to establish the importance of such disease markers or effectors.

Sample preparation is a critical step in the proteomics workflow as the quality and reproducibility of protein extraction and handling significantly impact coverage and quantitative analysis of the native proteome. Minimising sample preparation avoids proteoform degradation and modification. Most of the prefractionation methods we assessed involved several steps and resulted in unexplained variations in analyses of the same samples, including substantial overlap of protein species (i.e., poor separation) between fractions (Figure 2, Figure 3, Figure 4, Figure 5 and Figure 6). In contrast, starting with 500 µg serum protein on 3–10 NL IPG strips in the first dimension followed by second dimension resolution on a large 7–20% gradient gel by SDS/LDS PAGE improved the resolution and detection of protein species (Table 2). Little is known about the mechanism by which LDS and SDS in combination contribute to protein resolution, although it is well documented that LDS promotes the solubilization and resolution of certain hydrophobic proteins, particularly under the temperature conditions used in our established protocol (i.e., 4°C) [47,48,49]. In this study, in particular, low molecular mass protein species were strongly enriched using a combination of SDS and LDS confirming what has been previously noted in the literature [50]. Furthermore, adding LDS pre- and post-second dimension of electrophoresis (i.e., in the equilibrating buffer, the gel matrix, and the 2D buffer system) enabled resolution of certain proteins not detectable when using SDS or LDS alone (Table 2). We also confirm the resolution of proteoforms using selective staining for phospho- and glycoproteins (Figure 10).

## 5. Conclusions

When selecting a prefractionation method to assist in sample preparation, it is imperative to assess the potential for loss of low abundance proteins; the possibility of carrier protein interactions with critical low molecular weight biomarkers is likely a serious issue hampering further advances. Most studies that have investigated the serum proteome in various disease conditions have not been able to successfully characterize potential biomarkers nor validate their results in a larger cohort. Simply, altering the native stoichiometry of the proteome components may yield a more complex series of issues than dealing directly with the inherent complexity of the sample. This is the fundamental basis for our focus on top-down proteomic analyses of native samples.

In terms of the current investigation, maternal serum samples were pooled from three individual patients in order to evaluate methods and develop an analytical process. The method developed in this study is now being routinely applied to clinical samples. We will be applying the approach defined here to further characterize and quantify protein species in a larger cohort of subjects to provide a better understanding of the maternal serum proteome. One of the potential limitations of the results presented here was the need to ‘over’-load the gel with 500 µg protein in order to assess lower abundance species (although this only amounts to an average of ~10 µL of serum). While this would not be an issue for several standard clinical samples (e.g., plasma, urine, saliva), it may not be feasible with regard to other health complications [5]. Importantly, the advantages of the approach defined here cannot be overstated: quantitative analysis of protein species in their native melieu (i.e., without exposure to fractionation and other chemical manipulations). It is expected that this represents an opportunity for broader use of this critical top-down approach to proteomic analyses of serum and other important clinical samples. We anticipate that this top-down 2DE approach will prove to be a powerful tool for quantitative, reproducible and thorough analyses of proteoforms, and thus imperative to assessing health, disease state and progression, as well as the identification of critical biomarkers.

## Figures and Tables

**Table 1:** Total protein species resolved by 2DE after various methods of fractionation.

Methods	Type of Gel	Gel %	Protein Conc.	Protein Species Detected
Native serum (Baseline for statistical comparison)	Mini	12.5%	100 µg	^†^ 367 ± 2
Ultra 3 h		12.5%	100 µg	424 ± 21
Trichloroacetic acid (TCA)		12.5%	100 µg	358 ± 6
Tx-114		12.5%	100 µg	415 ± 3
Size exclusion filters		Frac A and Frac B (7–10%)	100 µg	^†^ 392 ± 24
		Frac C (12–20%)		
Aurum Affi-Gel Blue column		12.5%	100 µg	285 ± 7
Tx-114	Large	7–20%	500 µg	^†^ 779 ± 51 *

Values given are average for total spot counts; all mean values were derived from combining fractions; *n* = 6 gels, except ^†^ *n* = 4, Statistical significance indicated as * *p* ≤ 0.001; One way analysis of variance (ANOVA), Tukey’s multiple comparison test.

**Table 2:** A comparison of the total number of protein species resolved using different 2DE and deep imaging techniques.

Method	Type of Gel	Gel %	Protein Concentration	Number of Protein Species Identified
Sodium dodecyl sulfate (SDS) (0.1%)	Large gel	12.5%	500 µg	709 ± 15
No gradient (Baseline for statistical comparison)				
SDS (0.1%)		7–20%	500 µg	864 ± 11 *
Lithium dodecyl sulfate (LDS) (0.1%)		7–20%	500 µg	870 ± 12 *
SDS (0.1%) + LDS (0.1%)		7–20%	500 µg	919 ± 15 *
Deep imaging		7–20%	500 µg	942 ± 7 *
SDS (0.1%) + LDS (0.1%)				

Values given are mean ± SEM for total spot counts; all mean values were derived from three technical replicates. Statistical significance indicated as * *p* ≤ 0.001; One way ANOVA, Tukey’s multiple comparison test.

## Supplementary Materials

Supplementary Materials are available online.
