# Supplementary material for: A Routine ‘Top-Down’ Approach to Analysis of the Human Serum Proteome
Source: Proteomes. 2017 Jun 6;5(2):13. doi: 10.3390/proteomes5020013 (PMC5489773; doi:10.3390/proteomes5020013)
Supplement: Supplementary file 1 [file proteomes-05-00013-s001.pdf]

Supplementary material:

Materials and suppliers

Electrophoresis consumables including tris-glycine, acrylamide, SDS buffer and Coomassie Brilliant Blue G-250 dye (CBB) were purchased from Ameresco (Solon, OH). Tributylphosphine, broad range non-linear 7 and 17 cm, three to ten IPG strips, Bio-lyte broad range carrier ampholytes (pH 3–10) and each of four Bio-lyte narrow range ampholytes (pH 3–5,6–8, 7–9 and 8–10) were purchased from Bio-Rad Laboratories (Hercules, CA). Protease inhibitor cocktail components, aprotinin, pepstatin, leupeptin and sodium orthovanadate, were purchased from Sigma-Aldrich (St. Louis, MO).

2 - Dimensional gel electrophoresis (2DE) [essentially according to [1, 2]]

In brief, this involved loading either 100 µg or 500 µg of protein onto 7 cm or 17 cm, respectively, 3-10 non-linear (NL) immobilised pH gradient (IPG) strips by passive hydration for 16 h at room temperature (RT), after first having reduced and alkylated the samples. Isoelectric focussing (IEF) was carried out at 17°C in the Protean IEF cell (BioRad). 2DE was carried out on 12.5% or 7-20% gradient acrylamide gels unless stated otherwise. Following 2DE, each gel was fixed in 300 mL of 10% (v/v) methanol and 7% (v/v) acetic acid for 1 h at RT with gentle mixing, and subsequently washed with milliQ water for three times 20 min. Resolved proteins are subsequently detected in-gel using the current gold standard protocol: colloidal CBB (cCBB) as a near-infrared dye [3]. cCBB-stained gels were destained with 0.5M NaCl and imaged on the FLA-9000 (GE healthcare, Little Chalfont, United Kingdom) at 685/>750 ex/em with a PMT setting of 600 V and pixel resolution set to 100 µm. Phospho and glyco gels were imaged on the FLA-9000 (GE healthcare, Little Chalfont, United Kingdom) at 532/580 ex/em and 510/520 ex/em repectively with a PMT setting of 500V and pixel resolution set to 100 µm. Analysis of 2DE gel images was carried out using Delta 2D software (version 4.0.8; DECODON, Gerifswald, Germany). Gels were resolved in parallel replicates for each sample type.

Ultracentrifugation

The supernatant fraction collected after the first ultracentrifugation step was concentrated to 500 µL using a protein concentrator spin tube (3,000 kDa cut-off; Merck Millipore, USA) by centrifuging at 1008 × g, 4°C for 30 min using a Hettitch Rotina 420R centrifuge. The pellet was solubilised in 150 µL of 2D buffer and 1 × PI. To ensure removal of salts, 4 M urea (3500 µL) was added to the 500 µL supernatant

fraction and this was then centrifuged at 1008 × g, 4°C, for 30 min; this washing process was repeated three times. The final concentrated sample was 500 µL. Protein estimation was performed (see Material and Methods for main article) for both supernatant and pellet fractions. This method was also repeated with ultracentrifugation extended to 16 h in the first step. Overlap between supernatant and pellet fractions have been represented as a fusion image for gels obtained following both 2 h and 16 h ultracentrifugation steps (Fig. 1).

Trichloroacetic acid (TCA) precipitation

The supernatant from the centrifugation process was kept aside on ice. The pellet was washed in 200 µL of ice cold acetone (100% (v/v)) and incubated at -20°C for 10 min. This was then centrifuged at 15,000 × g for 15 min at 4°C. The supernatant was collected and combined with the first supernatant. The pellet was air dried in a fume hood at RT to drive off acetone and then solubilized in 2D sample buffer and 5 µL 5% DTT; protein estimation was performed on both fractions prior to 2DE.

Triton X-114: hydrophobic-hydrophilic phase separation

Following phase separation, the aqueous phase (AP) was removed from the tube without disturbing the lower detergent phase (DP), which was kept aside on ice. The AP received 1 mL of fresh 0.5% TX-114. The mixture was again overlaid on a sucrose cushion, incubated 3 min at 30˚C for condensation, and then centrifuged (all as above). This final AP was removed and rinsed with 2% Triton X-114 in a separate tube without the sucrose cushions. The DP from the second condensation was discarded. Proteins in both the AP and DP were precipitated using TCA (as above) and were initially analysed by sodium dodecyl sulfate polyacrylamide gel electrophoresis on mini gels [4]. The experiment was repeated after incorporating three AP wash steps with 10 mM Tris-HCl (pH 7.4), 150 mM NaCl in the final stage. Resulting DP and AP fractions were resolved on large 7-20% gradient gels. Fig 2 presents the overlap of proteins in the aqueous and detergent phases, respectively.

Size exclusion filters

Overlap in the distribution of proteins in fraction A (>100 kda) and fraction B (50-100 kda) is shown in Fig. 3.

Aurum Affi-Gel Blue column

According to the manufacturer's instructions, 125 µL of serum was diluted with 375 µL of the 'low-salt application buffer.' 400 µL of the diluted serum sample was loaded on the resin bed column and centrifuged for 20 sec at 10,000 x g collecting the residual eluate in the "unbound" collection tube. The resin was washed with 400 µL of the low-salt application buffer and the column was centrifuged as above collecting the eluate in the same "unbound" tube, which contained the albumin-depleted serum sample. The bound albumin was recovered from the Affi-Gel Blue column by eluting the column with 500 µL of 2DE buffer. Bound and Unbound fractions were analysed by 2DE, essentially according to [1, 2] (see Materials and Methods).

Lithium Dodecyl Sulfate (LDS) vs. Sodium Dodecyl Sulfate (SDS)

Briefly, using a standardized protocol [1,2], 500 µg of protein extract was loaded onto a 17 cm, 3-10 NL IPG strip by passive hydration for 16 h at RT, after having first been reduced and alkylated. These samples were then resolved on 7-20% gradient acrylamide gels. Following 2DE, the gels were fixed and imaged (see main text).

Results:

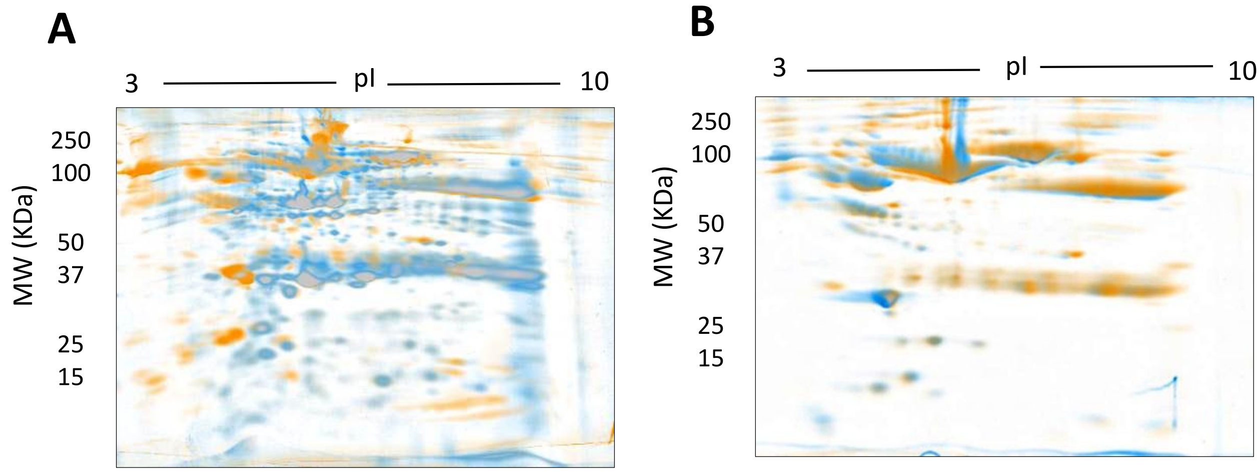

Figure 1: A is the fusion of supernatant (blue) and pellet (orange) proteome images after 2 h, and B is the fusion of supernatant (blue) and pellet (orange) proteome images after 16 h of ultracentrifugation.

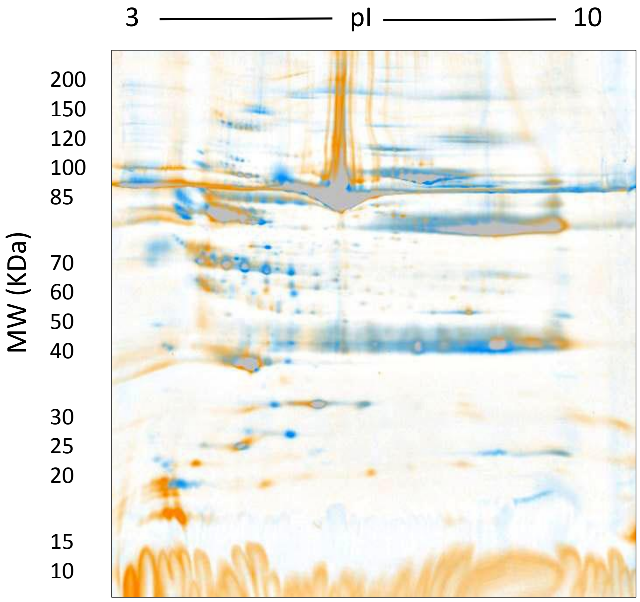

Figure 2: TX-114: Overlap of protein profile in the aqueous (blue) and detergent phases (orange).

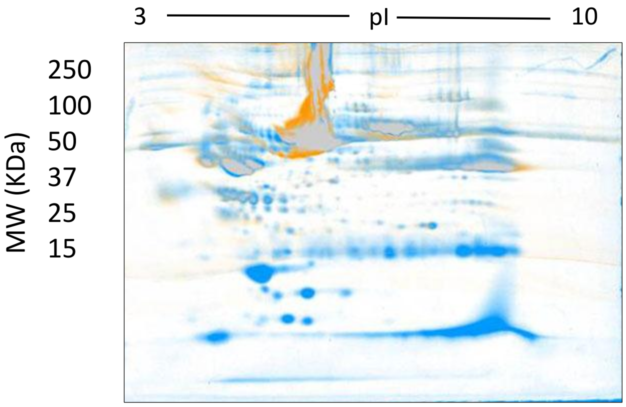

Figure 3: Fusion of >100 kda fraction A (blue) and the 50-100 kda fraction B (orange) proteome images following the use of size exclusion filters.

Table 3: Supplementary data

Methods	Protein species detected		Type of gel	Protein conc
Ultra 3 h	Membrane: 395 ± 5	Soluble: 454 ± 13	Mini (12.5%)	100 µg
Ultra 16 h	†Membrane: 314 ± 11	†Soluble: 220 ± 13	Mini (12.5%)	100 µg
TCA	Supernatant: 358 ± 6	Pellet: NA	Mini (12.5%)	100 µg
TX-114	AP: NA	DP: 415 ± 3	Mini (12.5%)	100 µg
TX-114	†AP: 708 ± 9	†DP: 851 ± 8	Large (7-20%)	500 µg
Size exclusion	†Fraction A: 364 ± 8	†Fraction B: 420 ± 10	Mini: Frac A (7-10%) Frac B (7-12%)	100 µg
	Fraction A: 580 ± 5	Fraction B: 335 ± 4	Large: Frac A (10%) Frac B (10%)	500 µg
Aurum Affi-Gel Blue column	Bound: 290 ± 7	Unbound: 280 ± 6	Mini (12.5%)	100 µg

Values given are mean ± SEM for total spot counts; all mean values were derived from three technical replicates except † resolved in duplicate.

References:

1. Butt, R.H. and J.R. Coorssen, *Pre-extraction sample handling by automated frozen disruption significantly improves subsequent proteomic analyses.* J Proteome Res, 2006. **5**(2): p. 437-48.
2. Wright, E.P., et al., *Top-down proteomics: enhancing 2D gel electrophoresis from tissue processing to high-sensitivity protein detection.* Proteomics, 2014. **14**(7-8): p. 872-89.
3. Gauci, V.J., M.P. Padula, and J.R. Coorssen, *Coomassie blue staining for high sensitivity gel-based proteomics.* J Proteomics, 2013. **90**: p. 96-106.
4. Churchward, M.A., et al., *Enhanced detergent extraction for analysis of membrane proteomes by two-dimensional gel electrophoresis.* Proteome Science, 2005. **3**: p. 5-5.
